# Prosthetic rehabiliatation of a hemimaxillectomy patient using a zygomatic - Corticobasal® implant- supported reconstructive prosthesis: A case report

**DOI:** 10.1016/j.ijscr.2025.110815

**Published:** 2025-01-03

**Authors:** Syed Akifuddin, Fadia Awadalkreem

**Affiliations:** a Consultant Oral and Maxillofacial surgeon and Implantologist, Dentomax Centre for Dentistry, Implants and Maxillofacial Surgery, Hyderabad, India; b Department of Prosthodontics, RAK College of Dental Sciences, RAK Medical and Health Sciences University, Ras Al Khaimah, United Arab Emirates

**Keywords:** Case report, Corticobasal® implant, Fixed implant supported prosthesis, Maxillectomy, Prosthetic rehabilitation, Zygomatic implants

## Abstract

**Introduction:**

Rehabilitation of patients with hemimaxillectomy presents a challenge. This case report describes the successful use of zygomatic Corticobasal® implant- supported reconstructed prosthesis.

**Clinical case presentation:**

A 20-year-old female patient presented to the clinic following hemimaxillectomy with soft tissue approximation one year ago. The patient was very depressed and reported high aesthetic concern and masticatory inefficiency and required a fixed prosthesis. A multidisciplinary team was formed. A panorama and cone beam CT were acquired. The treatment plan included the construction of an immediately loaded, fixed implant-supported reconstructive prosthesis using 6 Corticobasal® implant (BCS® and ZDI® implant designs, Dr. Ihde Dental AG, Switzerland) and a follow up program. After 3 years in function, the patient presented with 100 % implant survival rate, no complaints, and reported great improvement in esthetics, speech, mastication, and quality of life.

**Discussion:**

The use of zygomatic Corticobasal® implants in this case provides the significant advantages of improving the prosthesis support and the utilization of the strongest zygomatic bone for implant anchorage. Moreover, the use of a metal framework for implant splinting and the monoblock design of the implant reduce the risk of implant/prosthesis overloading, and eliminate the biomechanical complication. Furthermore, the provided fixed prosthesis matched the patient's desire and significantly optimized the patient satisfaction and quality of life.

**Conclusion:**

Within the limitation of the study, Corticobasal® implants can be used for rehabilitating hemimaxillectomy patients with optimum peri-implant soft tissue results, reducing risk of infection, achieving high survival rate and significantly improving the patient's aesthetic, functional, and satisfaction.

## Highlights

• Rehabilitation of patients with hemimaxillectomy presents a challenge.
• Corticobasal® implants can be used for rehabilitating hemimaxillectomy patients.
• It provides a fixed prosthetic option with optimum peri-implant soft tissue health.
• It significantly improves the patient's aesthetic, function, satisfaction, and quality of life.

## 1 Introduction

According to the glossary of prosthodontic terms, maxillectomy, or maxillary resection, is defined as the surgical removal of part or all of the entire maxilla [1,2].

Rehabilitation of patients with maxillectomy presents a challenge for both maxillofacial surgeons and prosthodontists. The aim of successful rehabilitation is to fulfil the following prerequisites: Closing the oroantral communication that could potentially compromise the health of the maxillary sinus [[3], [4], [5], [6]]. Preserve the orbital content and maintain the eyelid functions [[3], [4], [5], [6]], ensure a clear patent nasal airway, replace the dentition, and provide a masticatory units, improving the patient's cosmetic, lip and check support, patient's phonation, and self-satisfaction [5,6].

Reconstruction of maxillectomy can be performed through both surgical and prosthetic techniques. Several surgically reconstructed flaps have been proposed and used commonly for maxillary reconstruction including: Fibula, scapular, and iliac crest flaps. Moreover, in less complex cases, soft tissue approximation, temporalis muscle and buccinator myomucosal flaps represent local solutions with a lower surgical burden for the patient [7,8].

Despite the surgical reconstruction's ability to seal the oroantral communication, maintain the orbital content, and supply the necessary backbone tissue, the masticatory function remains impossible without a prosthetic replacement for the resected dentition [5]. These can be achieved through both removable and fixed prostheses supported by either teeth or implants [5].

Nowadays, the use of implant-supported prosthesis has been highlighted to improve the prosthesis's retention, support, and stability and to provide the patient with a fixed treatment modality [5,[9], [10], [11], [12]].

BCS® and ZDI® implants are special Corticobasal® implants charactrized by smooth surface with variable lengths range between 8–38 mm and 35–70 mm respectively. Moreover, ZDI® implants are used for zygoma and glabella anchorage. It's a one-piece implant that can be inserted using flapless or flap technique from the crestal direction targeting the second cortical or even the third cortical layers of the basal bone ie: Pterygoid Plate, Sphenoid bone and Zygomatic bone [13] a characteristic that qualified this implant to be uses in maxillofacial patients with a high success rate. Several investigators [[14], [15], [16], [17]] have reported the long-term sucessful use of Corticobasal® implant in maxillofacial patients, including mandibulectomy cases; however, there is still a lake of knowledge regarding their use in patients with maxillary defects.

This case report describes the successful use of Corticobasal® implant- supported reconstructed prosthesis with 3 years of follow-up. Ethical approval for the study and informed consents were obtained for treatment and publication. The case was in line with SCARE guidelines [19].

## 2 Case presentation

**Fig. 1:**
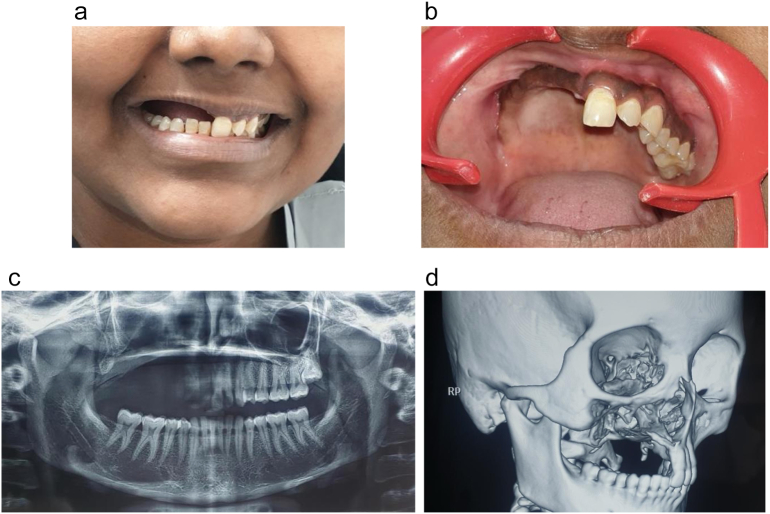
The patient's clinical presentation. A. The extraoral view of the patient. B. The intraoral view displays the patient's right partial maxillectomy following soft tissue approximation and healing. C. A panoramic radiograph revealing the right maxillectomy with complete mandibular and left-side dentition. D. A 3D Cone beam CT showing the maxillary defect.

A 20-year-old female patient presented to the clinic following hemimaxillectomy owing to tumor excision accompanied with soft tissue approximation one year ago (Fig. 1A, B). The patient had no family or allergy history and was very depressed, expressed high aesthetic concerns, reported masticatory inefficiency, and required a fixed prosthesis.

A multidisciplinary team was formed, including an expert oral maxillofacial surgeon and prosthodontists. Preoperative radiographs ie. Digital panorama and cone beam CT (Planmeca Pro Max, Finland) were acquired in order to evaluate the different rehabilitation options and identify the possibility of implant treatment (Fig. 1C, D). All the treatment options were discussed with the patient; the patient refused bone grafting procedure and insisted on the fixed prosthetic option; hence, the treatment plan included the construction of an immediately loaded, fixed, Corticobasal® implant- supported reconstructive prosthesis. The treatment plan was fully discussed with the patient, and informed consent was obtained.

**Fig. 2:**
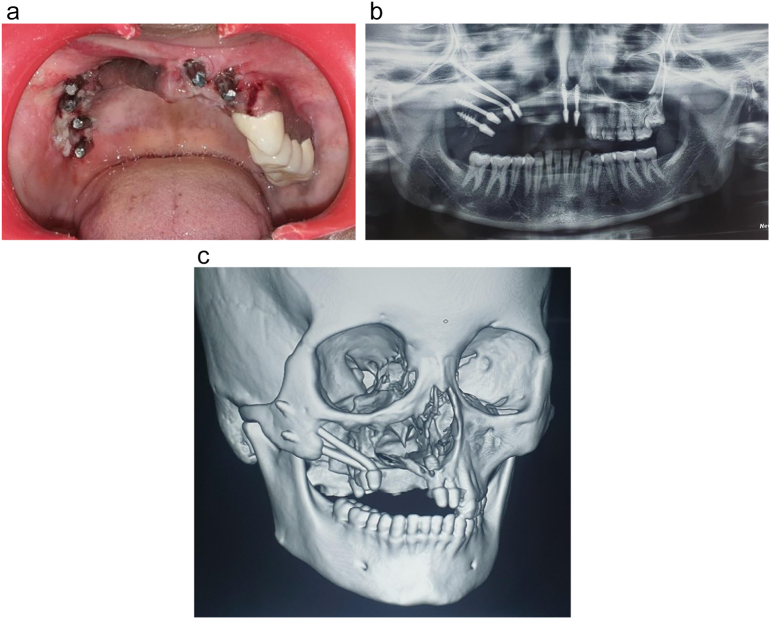
A. An intraoral clinical photograph presenting the distribution of the Corticobasal® implants. B. A panoramic radiograph revealing the implant distribution: one implant in the midline nasal bone, one implant in the contralateral nasal floor, two pterygoid implants, and two ZDI® implants in the zygomatic bone. C. A 3D Cone beam CT showing the Corticobasal® implants distribution.

Six Corticobasal® implants (BCS® and ZDI® implant designs, Dr. Ihde Dental AG, Switzerland) were inserted under an aseptic condition using local anesthesia (2 % lidocaine with epinephrine 1: 100,000). Implants were distributed as follows: one implant in the midline nasal bone, one implant in the contralateral nasal floor, two pterygoid implants, and 2 implants in the zygomatic bone (Fig. 2A).

Postoperative panoramic and cone beam computed tomography views were captured to verify the implant's positions (Fig. 2B, C). Impression was taken using monophase vinyl polysiloxane (VPS, Ivoclar Vivadent AG). Amoxicillin and clavulanate potassium 1 mg (Megamox, HIKMA), diclofenac potassium 50 mg (Rapidusk), and xylometazoline adult nasal drops (Otrivin, GlaxoSmithKline) were prescribed.

**Fig. 3:**
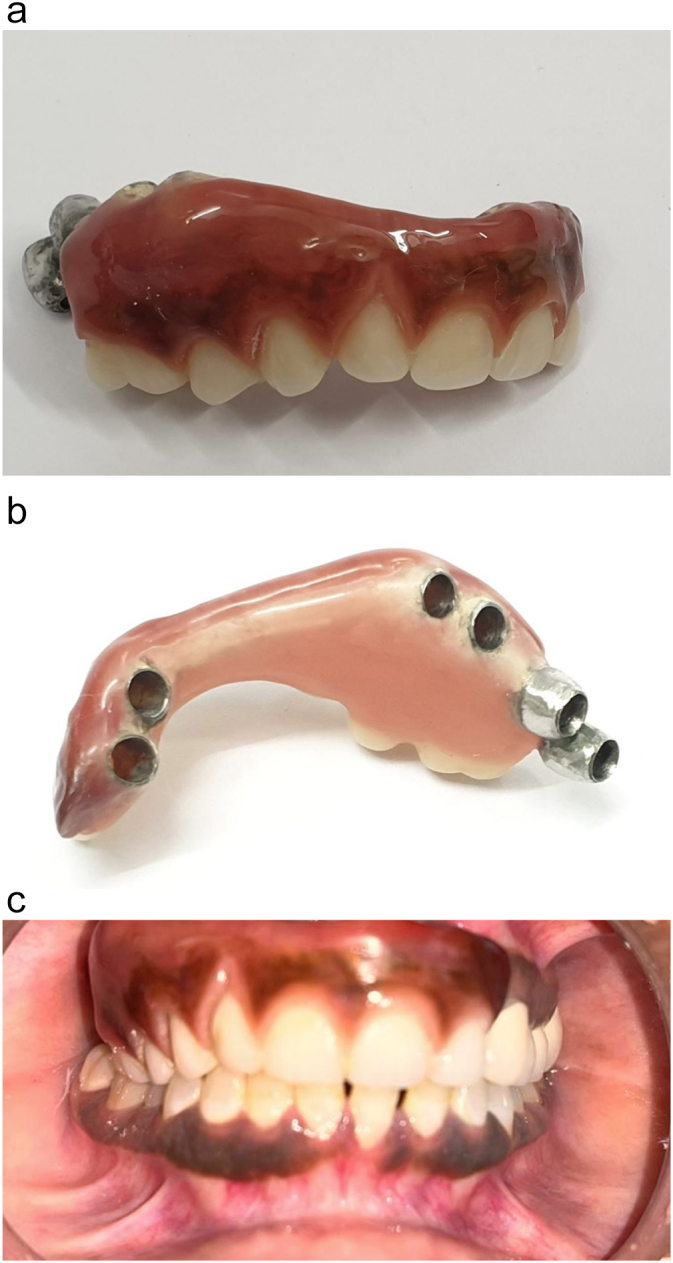
The prosthetic rehabilitation of the patient. A. An extraoral view of the final reconstructive prosthesis before insertion from the polishing surface. B. An extraoral view of the final reconstructive prosthesis before insertion from the fitting surface. C. The intraoral view displays the final fixed, immediately loaded, Corticobasal® implant- supported reconstructive prosthesis.

One day later, a metal framework try-in was performed, followed by silicone jaw relation. The contralateral teeth were crowned to elevate the patient's bite, improve the patient vertical dimension, face height, and aesthetic. On the third day, a hybrid Fixed Corticobasal® Implant Supported-Prosthesis was inserted and cemented using Fuji cement (GC Fuji I Luting Cement, Japan). The labial and palatal extension of the prosthesis was shorted to provide a hygienic space. The occlusal was checked and occlusal adjustment was performed to eliminate any deflective occlusal contact (Fig. 3A, B and C).

**Fig. 4:**
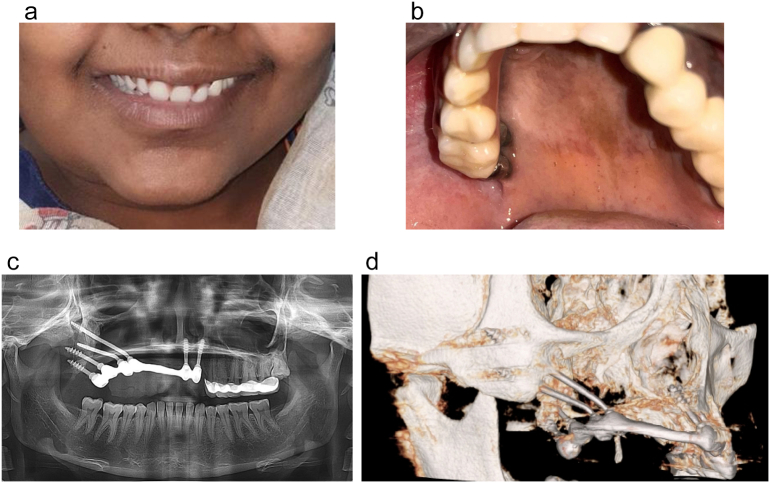
The patient's follow-up after 3 years of follow-up A. An extraoral view of the patient. B. The intraoral view of the patient depicting his clinical presentation exhibiting optimal peri-implant oral health kindly notes the smooth surface of the implant without plaque adherence. C. A panoramic radiograph after 3 years of function. D. A 3D Cone beam CT showing the prosthesis after 3 years of function.

The patient was scheduled for follow-up after 1 week and 3, 6, 9, 12, and 18 months and 6 months, therefore. At each follow-up, the patient was examined both clinically and radiographically, and occlusal adjustment was performed as needed. At 3 years of follow up, the patient presented with a 100 % implant survival rate, no complaints, and reported great improvement in her esthetics, speech, mastication, and quality of life (Fig. 4A, B and C).

## 3 Discussion

The ultimate goal of maxillary reconstruction is to restore the oroantral communication, to preserve the orbital content, and to improve the patient's aesthetic, function, and quality of life by providing the patient with a retentive and stable prosthesis [8].

The success of the treatment depends mainly on the practitioner's skill and judgement and is governed by the location and size of the defect, the quantity and nature of the remaining soft and hard tissue, the depth of the vestibule, the oroantral communication status, the use of radiotherapy and/or chemotherapy, and the patient preference [3,5]. In the prescribed case, despite the fact that the soft tissue approximation facilitates the closure of the oroantral communication, it adversely affects the depth and vestibule and eliminates the undercut used for removable obturator's retention. Additionally, it compromised the prosthesis support and stability and highlighted the use of implant-supported prostheses. An observation is that in line with many investigators [[20], [21], [22]]. On the other hand, the age, the high demand for fixed prosthesis, and the psychological status of the patient make the selected implant-supported reconstructive prosthesis the treatment of choice. Additionally, the multidisciplinary team approach significantly optimizes the treatment outcomes [15,18].

The use of Corticobasal® implants (BCS® and ZDI®) in this case provides the significant advantages of eliminating the need for bone grafting and subsequent risks [15], improving the prosthesis support, and the utilization of the strongest zygomatic bone for implant anchorage. According to the literature, the drawbacks of zygomatic implants include risk of infection, i.e., peri-implant soft tissue and sinusitis, and prosthesis overloading [5,10]. However, the selected implant system is characterized by a smooth surface that eliminates the risk of plaque retention, subsequent infection possibility, and ensures healthy peri-implant and sinus health. An observation that is in line with Ihde [13], Lazarov [23], and Awadalkreem et al. [15].

Moreover, the use of metal framework splinting for the implant, the monoblock design, as well as occlusal adjustment reduce the risk of implant and prosthesis overloading and ensure better force distribution and eliminate the biomechanical complication as reported by Misch et al. [24,25], Awadalkareem et al. [15,17], and Ihde et al. [13].

The high survival rate reported in this case matched the basal cortical screw implant survival rate documented by many investigators, including Awadalkree et al. [15] who reported an optimum implant health associated with 100 % survival and success rates with none of the implants being mobile, lost, or fractured. Vitomir et al. [16] reported a 90.3 % implant survival rate over 12 years of follow-up when Corticobasal implants used for the retention of extraoral prostheses. Goiato et al. [10] reported a survival rate of 97.86 % after a 36-month follow-up for zygomatic implants. A result that is in line with Aalam et al. [26] and Gulia S and Vigarniya MM [27].

The use of a fixed treatment modality in accordance to the patient's desire optimizes the treatment outcome and increases the patient satisfaction level. Additionally, the significant improvement in the aesthic, functions, and quality of life reported by the patient is in line with many investigators, including: Takaoka et al. [28], Karayazgan-Saracoglu et al. [29], and Yusa et al. [30].

The study's limitations include a limited sample size, but its strengths are highlighted by the optimal results, high survival rate, and patient satisfaction.

## 4 Conclusion

Within the limitation of the study, the Corticobasal® implants can be used for rehabilitating maxillectomy patients with optimum peri-implant soft tissue results, reducing or even eliminating the risk of infection, achieving a high survival rate, and significantly improving the patient's aesthetic, functional, and satisfaction outcomes.

## Author contribution

Akifuddin S contributed to the conceptualization, treating the patient, writing, editing, finalization of the case.

Awadalkreem F contributed to the conceptualization, treating the patient, writing, editing, finalization and submission of the case.

## Consent

Written informed consent was obtained from the patients for publication and any accompanying images. A copy of the written consent is available for review by the Editor-in-Chief of this journal on request.

## Ethical approval

The research had been approved by the ethical committee of Dentomax Centre for Dentistry.

## Guarantor

Fadia Awadalkreem.

## Funding

The authors claim to have no financial interests, either directly or indirectly, in the products or information listed in the article.

## Registration of research studies

The research was approved by the he ethical committee of Dentomax Centre for Dentistry and registered at the Research Registry with the unique identifying number: 10881. https://www.researchregistry.com/browse-the-registry#home/.

## Conflict of interest statement

The authors declare no conflicts of interest in connection with this research and manuscript.
